# Microbiota-Derived Extracellular Vesicles as New Systemic Regulators

**DOI:** 10.3389/fmicb.2017.01610

**Published:** 2017-08-24

**Authors:** Sara Ahmadi Badi, Arfa Moshiri, Abolfazl Fateh, Fatemeh Rahimi Jamnani, Meysam Sarshar, Farzam Vaziri, Seyed Davar Siadat

**Affiliations:** ^1^Mycobacteriology and Pulmonary Research Department, Pasteur Institute of Iran Tehran, Iran; ^2^Microbiology Research Center, Pasteur Institute of Iran Tehran, Iran; ^3^Department of Biology, Science and Research Branch, Islamic Azad University Tehran, Iran; ^4^Department of Biotechnology, School of Advanced Technologies in Medicine, Shahid Beheshti University of Medical Sciences Tehran, Iran; ^5^Experimental Therapy Unit, Laboratory of Oncology, G.Gaslini Children's Hospital Genoa, Italy; ^6^Laboratory affiliated to Instituto Pasteur Italia-Fondazione Cenci Bolognetti, Department of Public Health and Infectious Diseases, Sapienza University of Rome Rome, Italy

**Keywords:** gut Microbiota, extracellular vesicles (EVs), leaky gut, inter-kingdom communication, gene expression regulation, systemic regulation

The gut microbiota and their products play a critical role in metabolism, neurologic status, endocrine and immune system. There are pieces of evidence that dysbiosis in the gut is a factor in an astounding array of conditions and diseases: irritable bowel syndrome (IBS), autism, asthma, cancer, obesity, diabetes, neurological disorders, cardiovascular, and fatty liver disease (Zeng et al., 2017).

Microbiota-derived extracellular vesicles (EVs) carry a large diversity of compounds that can affect diverse pathways in the host. We propose to use various EVs not only as adjuvants for vaccination (Moshiri et al., 2012) but also as potential modifiers of host interactions in the gut that in turn affect several organ functions such as alteration of signaling molecules at the intestinal barriers (Cañas et al., 2016).

## The orchestra of gut microbiota in health and diseases

The number of bacteria in the human body is about 3.8^*^10^13^, which is approximately equal to 10 times the number of our total cells (Sender et al., 2016). Furthermore, the whole genome of microbiota include 150- to 400-fold more genes than the human genome. The gut microbiota has been developed in the presence of host cells and has demonstrated significant effects on host responses (Lloyd-Price et al., 2016).

Gut microbiota is key regulators of digestion. In the gut, the digestive slurry contains essential commensal bacteria that facilitate digestion, absorption, and synthesis of various metabolites, such as short-chain fatty acids (SCFAs). These metabolites produced by gut microbiota are directly linked to environmental factors like diet and life style. Therefore, they considered being diet-dependent metabolites (Brestoff and Artis, 2013). In addition, gut microbiota generates various diet-independent metabolites which include lipopolysaccharide (LPS) and peptidoglycan (PG) as well as EVs (Caricilli and Saad, 2013).

In the intestinal immune system maintaining homeostasis is challenging (Van Den Elsen et al., 2017). To perform this task, it is essential for the gut microbiota to learn how to communicate with epithelial and immune cells of the gastrointestinal tract (GIT). The interaction between gut microbiota and the host involves a complex network of signaling pathways (Thompson et al., 2015).

## Decoration of extracellular vesicles

All eukaryotic and prokaryotic cells release vesicle structures containing cytosolic and membrane proteins, phospholipids, glycolipids, polysaccharide, and nucleic acids referred as EVs. These vesicles have several functions concerning intercellular communication and signaling events. EVs refer to outer membrane vesicles (OMVs) or membrane vesicles (MVs) in Gram negative and Gram positive bacteria, respectively. OMVs and MVs have important biological roles not only in bacterial survival but also in host interaction, by in intra and inter-kingdom communication without intimate intercellular contact (Fateh et al., 2015).

The characterization of EVs biological roles has become a fascinating research field due to their potential properties in vaccination, therapeutic applications as vehicle in drug delivery and target therapy, positional assembly of enzyme expression and their ability in carrying small molecule activators and inhibitors of quorum sensing (Moshiri et al., 2012; Badmasti et al., 2015).

Fascinatingly, EVs carrying quorum-sensing molecules and bioactive compounds were able to participate as activators and inhibitors of quorum sensing involved in cell-cell communication. Furthermore, the DNA found in the EV has been successfully transferred into other bacterial cells, so this procedure may constitute a new DNA delivery system (Simonov et al., 2016).

EVs may display several bacterial cell surface components such as outer membrane proteins (OMPs), lipids, LPS, PG, and also carry different bacterial components such as DNA, RNAs, enzyme, toxin, and a complex of microbial associate molecular patterns (MAMPs) (Fateh et al., 2015). So a wide variety of pattern recognition receptors (PRR), such as Toll-like receptors (TLRs) and Nod-like receptors (NLRs), could be stimulated and activated by different EVs. TLRs trigger NFκB, mitogen-activated protein kinase (MAPK) and the interferon signaling cascades that affect the expression of interferons, related cytokines, chemokines, and antimicrobial molecules (Bielig et al., 2011; Kaparakis-Liaskos and Ferrero, 2015; Koeppen et al., 2016).

## Microbiota-derived EVs

The role of microbiota-derived EVs has received huge attention in recent years. This role is not only focused on their ability to carry a wide variety of digesting enzymes but also emphasized to immunomodulation and signaling pathways (Fábrega Fernández et al., 2016). For example, *Bacteroides fragilis*-derived EVs carrying polysaccharide A delivered to intestinal dendritic cells and could be sensed by TLR2, and also *Bacteroides thetaiotaomicron* EVs contain hydrolytic enzymes that increase the potential digestion of gut microbiota by sharing these enzymes with bacteria lacking the hydrolytic enzymes (Shen et al., 2012).

Therefore, the administration of bacterial specific strains derived EVs may modulate immune signaling pathways, host nutrition, and bacterial metabolites production (Shenderov, 2013). The EV-based network likely represents important relationships creating organized ecological units within the intestinal microbiota (Mitsuhashi et al., 2016).

## What is the scenario?

In a healthy state, the pattern and the function of the gut microbiota are stable. The gut barrier function is maintained via several mechanisms such as the appropriate localization of tight junction proteins, a normal endocannabinoid system tone and LPS detoxification by intestinal alkaline phosphatase. Altogether, these factors maintain appropriate energy, lipid and inflammatory homeostasis (Tremaroli and Bäckhed, 2012). Given the fact that, different environmental factors and aging could influence the composition of the gut microbial community, therefore, not only in homeostasis but also in dysbiosis of gut microbiota, EVs production may change and differ according to different gut ecology status. Therefore, regarding the effective roles of EVs, any changes in EV production can influence the host signaling pathways (Li et al., 2016). In dysbiosis conditions, the gut microbiota-host interactions will be impaired which in turn promote many diseases such as obesity and type 2 diabetes. This increase in gut permeability results from different disturbances such as alterations in the gut microbiota composition and/or their activities. Gut barrier alterations are responsible for metabolic endotoxemia leading to low-grade inflammation and metabolic disorders (Everard and Cani, 2013).

According to the complexity of gut microbiota and its interaction with the host, it is crucial to investigate the immune system and structures of microbiota which have a regulative character (Thaiss et al., 2014). In some diseases, disrupted normal immune system causes malfunction of gut barrier that results in the unusual transition of gut microbiota through the gut epithelium. In a recent study, induction of the leaky gut in animal models made the intestinal mucosal layer fine and permeable and provided a possibility for bacteria to infiltrate into the gut barriers. In this condition, *Akkermansia muciniphila* could not play as a detrimental microorganism to induce a protective effect. Instead, application of *A. muciniphila*-derived EVs impaired the progression of the disease (Kang et al., 2013; Fábrega Fernández et al., 2016). Therefore, we propose that in leaky gut syndrome, the probable harmful effect of gut microbiota could be replaced and inverted by gut microbiota-derived EVs treatment.

## Does any of EVs have priority to be called beneficial?

In a fascinating study, Kang et al. (2013) reveal the important effects of gut microbiota EVs on the intestinal immunity and its regulation. In disrupted barrier conditions such as experimental colitis, EVs of some bacteria, like TM7, a candidate bacteria phylum, increased suggesting their pathogenic characteristic with a direct or indirect effect on disease.

On the contrary, *A. muciniphila* and *Bacteroides acidifaciens*-derived EVs decreased. However, EVs produced by *A. muciniphila* demonstrated to ameliorate disease severity (Kang et al., 2013). In another study, *B. fragilis*–derived OMVs showed to be able to mediate anti-inflammatory responses by delivering Polysaccharide A to Dendritic cells expressing TLR2 (Shen et al., 2012).

## EVs may modulate gut-brain axis (GBA)

In recent years, the effects of gut microbiota on the brain and vice versa are proven. Also, the gut microbiota affects other communication pathways between the gut and the brain, including the nervous, immune and endocrine systems (Van Den Elsen et al., 2017).

New studies revealed that bacteria EVs are able to enter the systemic circulation and pass through blood brain barrier. Since EVs, that contain MAMPs, RNA, and DNA, could have the same effect of gut microbiota in GBA. Therefore, the application of EVs may have a considerable effect on GBA (Kelly et al., 2015; Muraca et al., 2015). This potential could be employed in gut-brain disorders such as IBS and psychopathic disorders.

## Can EVs affect epithelial-mesenchymal transition (EMT)?

The ability of epithelial cancer cells to convert to more motile form (Mesenchymal) is referred as EMT. Actually, this process helps epithelial cancer cells to spread from primary site to other organs (metastasis). Many factors could have the effect on EMT, such as cytokines (Valdez et al., 2016; Ortiz-Montero et al., 2017). Reversing this event (MET: Mesenchymal-Epithelial Transition) could be valuable as an anti-metastatic approach (Lamouille et al., 2014). We propose the EVs application may affect EMT or MET via indirect effects. If we accept that EVs can regulate cytokine genes expression, it may indirectly activate EMT or MET.

## Could mapk signaling associate to EVs research?

The MAPK signaling pathway is activated by a number of extra and intracellular stimuli including cytokines, growth factors, and hormones as well as stressors such as microbial-elicited reactive oxygen species (ROS). Recent data have demonstrated that gut epithelia in contact with gut microbiota rapidly generate ROS. While production of ROS by phagocytes in response to pathogens or host microbiota is supposed to be an important feature, other cell types, like intestinal epithelia, similarly have the potential to generate ROS in response to environmental signals. This pathway plays a key role in the regulation of many cellular processes including proliferation, differentiation, the stress response, motility, growth, differentiation, survival, apoptosis, and death (Kaparakis-Liaskos and Ferrero, 2015). In this regard, we propose the possible role of gut microbiota-derived EVs to mediate regulation of ROS production through its enzymes.

## Regulation of gene expression might be modified by EVs

Both eukaryotic and prokaryotic cells can deliver functional molecules to distant extracellular compartments and tissues. Exosomes, eukaryotic-derived vesicles, are involved in the horizontal transfer of genetic material, transport of mRNAs, and miRNAs to host cells. In addition, host genes that deposit lipids in adipocytes are linked with the alteration of gut microbiota composition. Gene expression of fasting-induced adipose factor (Fiaf), a lipoprotein lipase inhibitor, could be suppressed by gut microbiota alteration. This leads to the triglycerides increase which is stored in the liver (Tremaroli and Bäckhed, 2012).

Celluzzi and Masotti emphasized that the gene expression of the intestinal tissue and the gut microbiota composition might be generally correlated (Celluzzi and Masotti, 2016). They also suggested that the relationships between non-coding RNAs (ncRNAs) and microbiota deserved more investigation in order to unravel their mutual role in influencing the host immune system and related processes (Celluzzi and Masotti, 2016). Bacteria might release some soluble factors other than toxins or other peptides that could function as a “communication vector” and small RNAs within the OMVs might be one of these vectors (Koeppen et al., 2016). Moreover, their data demonstrate that the RNA found in OMVs aligned to some of the host chromosomes, thus leading to the conclusion that OMVs might be able to affect epigenetic processes (Celluzzi and Masotti, 2016).

## Concluding remarks

According to recent studies, EVs are able to deliver MAMPs, bound or soluble, to the host cell, leading to the activation of PRRs (Bielig et al., 2011). Unraveling how EVs control immune responses through alteration in their MAMPs contents, and how they are able to affect different signaling pathways in the host cells and organs show the importance of manipulated EVs production and to optimize their desired characteristics. Here, we have reviewed the recent advances and studies of EVs shed by bacteria, focusing on their inter-kingdom signaling role and their potential properties in the gut microbiota-eukaryote interaction that could be exploited in a near future as an alternative to pro-/pre-biotic supplementation.

## Author contributions

SS, SA, and AM contributed concepts and ideas and drafted the initial manuscript. SS, SA, AM, AF, FV, and FR reviewed articles and edited the manuscript. All authors commented on the final version of the manuscript.

### Conflict of interest statement

The authors declare that the research was conducted in the absence of any commercial or financial relationships that could be construed as a potential conflict of interest. The reviewer AC and handling Editor declared their shared affiliation, and the handling Editor states that the process nevertheless met the standards of a fair and objective review.
